# ISCEV standard for clinical multifocal electroretinography (mfERG) (2021 update)

**DOI:** 10.1007/s10633-020-09812-w

**Published:** 2021-01-25

**Authors:** Michael B. Hoffmann, Michael Bach, Mineo Kondo, Shiying Li, Sinead Walker, Karen Holopigian, Suresh Viswanathan, Anthony G. Robson

**Affiliations:** 1 Department of Ophthalmology, Otto-von-Guericke University, Magdeburg, Germany; 2 Center for Behavioral Brain Sciences, Magdeburg, Germany; 3 Eye Center, Medical Center – University of Freiburg, Faculty of Medicine, University of Freiburg, Freiburg, Germany; 4 Department of Ophthalmology, Mie University Graduate School of Medicine, Tsu, Mie Japan; 5 Department of Ophthalmology, Xiang’an Hospital of Xiamen University, Medical Center of Xiamen University, School of Medicine, Xiamen University, Xiamen, China; 6 Eye Institute of Xiamen University, Xiamen, China; 7 Glasgow Centre for Ophthalmic Research, Gartnavel General Hospital, Glasgow, UK; 8 Novartis Institutes for BioMedical Research, Novartis Pharmaceuticals, East Hanover, NJ USA; 9 State University of New York College of Optometry, New York, USA; 10 Department of Electrophysiology, Moorfields Eye Hospital, London, UK; 11 Institute of Ophthalmology, University College London, London, UK

**Keywords:** Clinical standards, Electroretinogram, Multifocal electroretinogram, International Society for Clinical Electrophysiology of Vision (ISCEV)

## Abstract

The multifocal electroretinogram (mfERG) is an electrophysiological test that allows the function of multiple discrete areas of the retina to be tested simultaneously. This document, from the International Society for Clinical Electrophysiology of Vision (ISCEV), presents an updated and revised ISCEV standard for clinical mfERG and defines minimum protocols for basic clinical mfERG recording and reporting so that responses can be recognized and compared from different laboratories worldwide. The major changes compared with the previous mfERG standard relate to the minimum length of m-sequences used for recording, reporting of results and a change in document format, to be more consistent with other ISCEV standards.

## Introduction

The full-field electroretinogram (ERG) is a mass potential, which reflects the summed electrical activity of the retina. Full-field electroretinography is a well-established clinical technique for evaluating global retinal function [1, 2]. The multifocal ERG (mfERG) was developed to provide a topographic measure of retinal activity and is widely used for clinical purposes [1, 3]. With this technique, many local cone-driven ERG signals, typically 61 or 103, are recorded from the retina under light-adapted conditions. This document updates the ISCEV Standard for mfERG testing and supersedes the 2012 version [3]. It defines minimum protocols for basic clinical mfERG recording and reporting so that responses can be recognized and compared from different laboratories worldwide. The major changes compared with the previous mfERG standard relate to the minimum length of m-sequences used for recording, reporting of results and a change in document format, to be more consistent with other ISCEV standards. Reports of clinical mfERG recordings performed to the standard method given here should cite this 2021 standard. Where a method is used which deviates from the standard method, the differences should be stated.

ISCEV publishes and maintains other standards for clinical electrophysiological testing: specifically for the full-field ERG [2], pattern ERG [4], electrooculogram [5] and visual evoked potential [6] as well as technical and calibration guidelines for clinical electrodiagnostic testing [7] and extended protocols [8–15]. The ISCEV web site should be consulted for current updates (www.ISCEV.org/standards). This document is not a safety standard, and it does not mandate particular procedures for individual patients or define the qualifications of those administering or interpreting the tests.

### Description of multifocal electroretinography

**Fig. 1:**
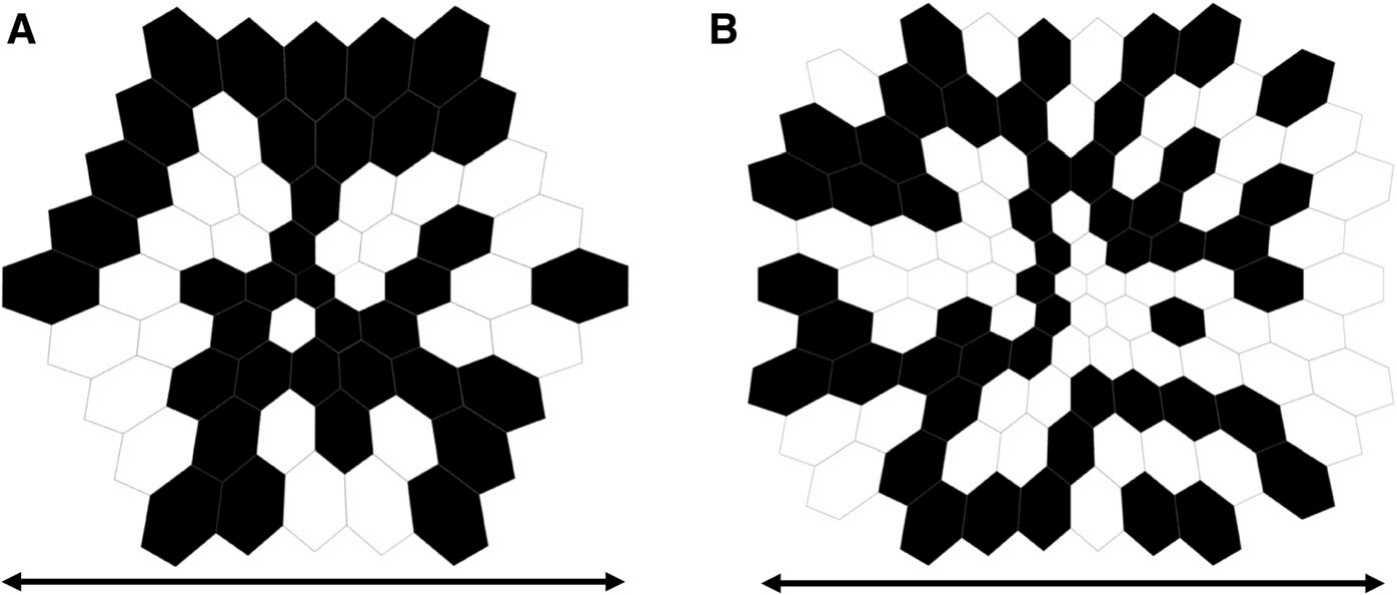
Typical mfERG-stimuli showing hexagonal frames scaled to be larger with increasing eccentricity and containing **A** 61 elements or **B** 103 elements. Individual hexagon outlines are added for clarity. The horizontal extent (arrows) of the stimulus array for standard mfERG recordings ranges between 40° and 50°

**Fig. 2:**
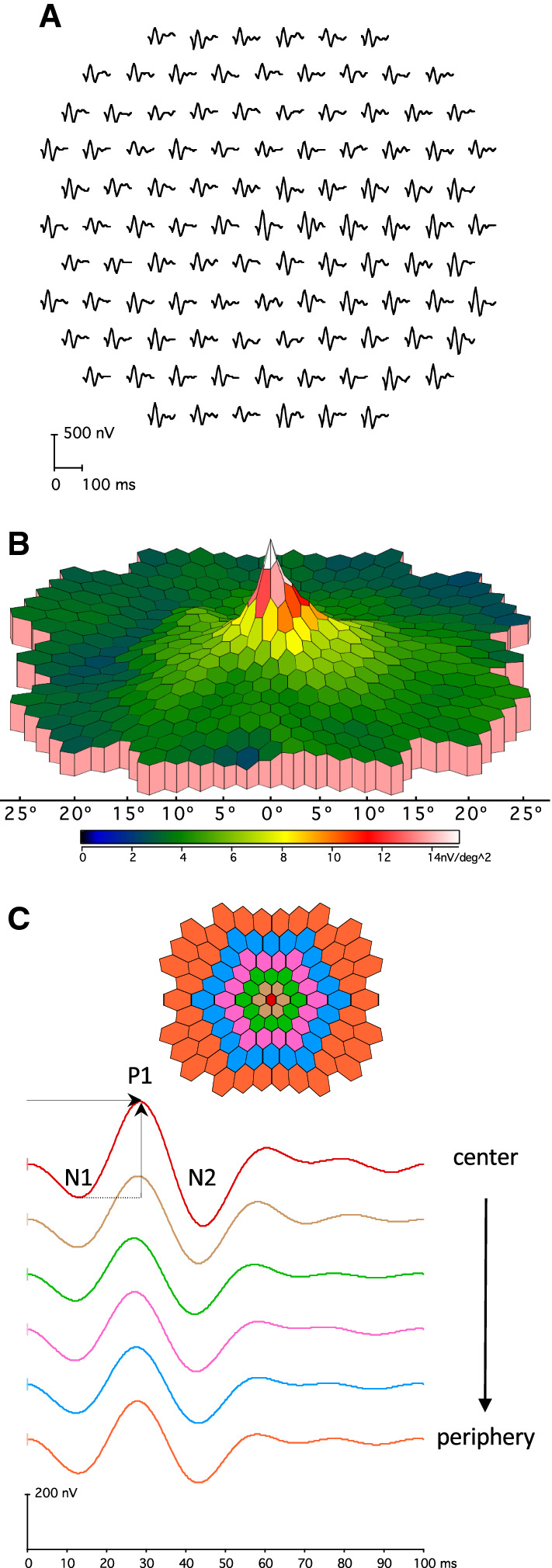
Sample mfERG recording obtained to a stimulus array containing 103 elements. **A** Traces (left eye; field view) from different eccentricities are arranged in an equidistant manner for clear visualization and comparison, while the actual stimulus array is scaled (see Fig. 1b). **B** 3D-response density plot (field view). Overall signal strength is given per unit area of retina. **C** Ring-averages. MfERG traces from the concentric hexagons were averaged within six different eccentricity ranges (see color coding in stimulus schematic) and arranged vertically from center to periphery. MfERG peak definitions (N1, P1, and N2) and P1-amplitude (trough to peak, vertical arrow) and P1-peak time (horizontal arrow) measures are indicated for the foveal response. The horizontal broken line corresponds to the trough of the N1 component

The mfERG technique allows recording of electrical signals from multiple discrete areas across the posterior pole, enabling the topographic representation and localization of retinal activity. Briefly, for the standard mfERG described here, stimuli comprise an array of 61 or 103 hexagons (Fig. 1). Each hexagon can take two states, light and dark, i.e., on and off. It changes rapidly between these two states, driven by a predetermined “pseudorandom” binary sequence (m-sequence). The m-sequences are identical for the different hexagons, but shifted in time relative to each other, and are mathematically independent (orthogonal). An automated cross-correlation of the recorded signal with the sequence of on/off stimulus states (m-sequence steps) for a specific hexagon allows for the extraction of the corresponding local ERG. Consequently and importantly, the local responses are not recorded directly at a specific retinal location, but are extracted from the continuous ERG signal, based on temporal characteristics. This distinction can be of importance for the interpretation of the mfERGs. The extraction of the standard mfERG signals associated with single illumination events is termed the first-order response or first-order kernel. For the extraction of the first-order kernel (K1), responses following a light stimulus step are added while those following a dark stimulus step are subtracted. Due to the rapid nature of the stimulus sequence and the way that signals are computed, additional information can be extracted by taking the stimulation history into account (higher-order responses/kernels) in non-standard mfERG applications. To avoid kernel overlap and to extract discrete kernels, a sufficiently long m-sequence is needed. The typical waveform of the standard mfERG comprises a biphasic wave with an initial negativity followed by a positivity and a negativity, termed N1, P1, and N2, respectively (Fig. 2C). It can be understood primarily as a combination of overlapping cone On- and Off-bipolar cell contributions combined with smaller contributions from cone photoreceptors. Although there are homologies between the mfERG waveform and the conventional full-field ERG, the stimulation rates are higher for the mfERG and the mfERG responses are mathematical extractions. Consequently, the mfERG responses are not “low-amplitude ERGs.” Therefore, the designations “a-wave” and “b-wave,” used for full-field ERGs, are not appropriate to describe features of the mfERG waveform.

### Clinical application of mfERGs

The mfERG is a clinical tool used to exclude, detect or characterize dysfunction over discrete retinal regions. A disease process that substantially reduces or delays mfERG N1 and P1 must be acting at, or before, the bipolar cells. The mfERG is typically employed to detect diseases of the outer retina affecting local function of cone-photoreceptors and bipolar cells.

A comprehensive list of clinical applications is beyond the scope of this standard, but typical examples include the investigation of central or paracentral maculopathies, assessment of dysfunction induced by hydroxychloroquine, assessment of posterior pole involvement in peripheral retinopathies such as retinitis pigmentosa and investigation of local retinal defects such as those associated with acute idiopathic enlarged blind spot syndrome (AIBSE) or acute zonal occult outer retinopathy (AZOOR). Some clinical examples are shown in the ISCEV guide to visual electrodiagnostic procedures [1].

## Basic technology

### Electrodes

#### Recording electrodes

Electrodes are required that contact the cornea, or nearby bulbar conjunctiva. This includes fiber, foil, loop and contact lens electrodes. The electrodes should allow for good retinal image quality and optimal refraction. The signal-to-noise ratio (SNR) of the responses is affected by the choice of the electrode. Bipolar corneal contact lens electrodes typically yield recordings with a high SNR; to obtain comparable SNRs with other corneal electrodes may require longer recording times, repeat measurements and/or fewer stimulus elements.

#### Reference and ground electrodes

Proper application of suitably conductive electrodes is essential for reliable mfERG recordings. Recordings are comparable only when the same electrode types and locations are used. Follow the recommendations made in the ISCEV Standards for full-field ERG [2] and pattern ERG [4].

#### Electrode characteristics, stability, and cleaning

Poor or unstable electrode contact is a major cause of poor-quality records. Follow the recommendations concerning fiber, foil, loop and contact lens electrodes in the ISCEV Standards for full-field ERG [2] and Pattern ERG [4]. Electrodes (if not disposable) must be suitably cleaned and sterilized after each use. The cleaning protocol should follow the manufacturers’ recommendations and meet current local and national standards for devices that contact skin and tears.

### Stimulation

#### Stimulus source

While in the past mfERG stimuli were commonly displayed on a cathode ray tube (CRT), they may now be generated on thin-film-transistor (TFT) -type liquid crystal displays (LCDs) and other displays, e.g., organic light-emitting diode (OLED) screens. These alternative modes of stimulation can affect the amplitude and waveform of mfERGs [16], making it essential to report the type of display and to specify the details of the manufacturer and model when reporting results.

#### Response time of displays

The time it takes for a local element (e.g., pixel) to go from dark to light and light to dark is termed response time. It determines the flash duration of a stimulus and must be sufficiently brief. This is not a problem with CRT monitors, which typically present, although not at exactly the same time across the field, a flash with a microsecond rise time followed by a 2 ms decay time. In contrast, TFT-type LCD panels typically switch between states and remain dark or bright for most of the frame and the response times of some of the displays can be longer than the duration of a single frame. Response times should be considerably less than the duration of an m-sequence step (e.g., for a step duration of one frame at 75 Hz frame rate <<13.33 ms). Response times and flash duration should be verified, e.g., by reference to the equipment-specific documentation supplied by the manufacturer.

#### Frame rate

Frame rates between 60 and 75 Hz are typically used. The use of different frequencies can substantially alter the amplitude and waveform of the mfERG. It is essential to be aware of the frame rate when interpreting results.

#### Luminance and contrast

The stimulus elements in the light state should be at least 100 cd/m^2^. The luminance of the display in the dark state should be low enough to achieve a Michelson contrast ≥ 90%. For all standard recordings, the luminance of the surround region of the display (the area beyond the stimulus hexagons) should approximate to the mean luminance of the stimulus array.

#### Calibration

As with other visual electrophysiological tests, luminance and contrast affect the recorded signals and it is important for the stimulus to be calibrated following ISCEV guidelines [7]. The luminance of the dark and the light stimulus elements should be measured with an appropriate calibrator or photometer. Many monitor screens are not of uniform brightness over the entire screen. While some variation is to be expected, a variation of greater than 15% is considered unacceptable. Some commercial systems are equipped to calibrate the display. If this facility is not available, device-specific manufacturer instructions may be needed.

#### Stimulus pattern

The standard stimulus field comprises an array of scaled hexagons (see Fig. 1 for examples) with a central fixation target. Stimulus parameters are specified in the following subsections addressing fixation target, size, number of elements and scaling. Different patterns may be useful in special cases (e.g., equal size hexagons for patients with eccentric fixation), but are beyond the scope of the current standard.

Fixation target. Stable fixation is essential for obtaining reliable mfERG recordings. Central fixation dots, crosses and circles are available with most commercial systems. The fixation target should cover as little of the central stimulus element as possible to avoid diminishing the response. At the same time, the examiner should always verify that the patient can see the fixation target or use cross hairs to help subjects to stabilize central fixation, e.g., in the presence of central scotoma. When the fixation targets are enlarged for low-vision patients, care should be taken not to obscure regions of interest. Such masking may lead to response attenuation and must be considered in comparison with reference data. Note that if the patient has good fixation in the fellow eye, the recording can be performed with both eyes open, although care is required in interpreting the results as there may be a misalignment between the two eyes (see “*Monocular versus binocular recording”*). If strabismus is present, recordings should be performed monocularly, with the fellow eye covered.

#### Size

For routine clinical examinations, the field should span a diameter of 40°–50° (20°–25° radius from the fixation point to the edge of the stimulus) as shown by the arrows in Fig. 1. It is also important to specify, or ideally show on trace arrays, the dimensions of the stimulus zone in degrees so that comparisons can be made to fundus images, visual fields and other measures of interest.

#### Number of elements

For routine clinical examinations, the field should contain either 61 or 103 hexagons. The choice of 61 versus 103 elements depends on balancing the need for good spatial resolution and a high SNR, while minimizing the recording time. Increasing the number of stimulus elements or decreasing the duration of the recording will decrease the SNR of the responses. Decreasing the number of elements will increase the SNR, but will decrease the spatial resolution of the test. For special applications, e.g., assessment of children, coarser stimuli (19 or 37 hexagons) may be useful, as precise fixation may be less critical and if reduced, small signals are more easily detected, but testing with fewer than 61 hexagons does not constitute a standard mfERG.

#### Scaling

The standard display is a hexagonal stimulus pattern that is scaled in size such that hexagons are larger with increasing eccentricity. This enables mfERGs of approximately equal amplitudes to be recorded across the healthy retina. Consequently, care should be exercised to avoid major eccentricity-dependent amplitude differences for the healthy retina.

#### Temporal sequence

In mfERG testing, m-sequences are used to control the temporal sequence of change between the two stages, light and dark, of each stimulus hexagon. These binary m-sequences are the standard for routine testing. To achieve sufficient SNR, the m-sequences should ideally have a length of at least 4095 steps (i.e., 2^12^–1 steps) for standard mfERG recordings with 61 or 103 hexagons. While different sequences, or the inclusion of global light or dark frames, have been suggested for specialized applications, such tests do not constitute a standard mfERG for routine clinical purposes.

### Recording and analysis

#### Amplifiers and filters

Patient isolation and input characteristics should follow the ISCEV ERG standard [2]. The gain of the amplifier should produce recognizable signals without saturation. Appropriate band-pass filtering removes extraneous electrical noise while it leaves the waveforms of interest largely undistorted. The preprocessing filtering is accomplished by the amplifier and, in some cases, by the commercial software. For a basic mfERG, the band pass of the filters should be approximately 5–200 Hz. The acceptable range for the high-pass cutoff is 3–10 Hz and for the low-pass cutoff is 100–300 Hz. Filter settings, even within these ranges, can markedly influence the response waveform. Thus, the filter settings must be the same as those used to record reference data and should be consistent if patients are tested repeatedly. Line-frequency or notch filters should be avoided and mains interference minimized at the source.

#### Signal analysis

##### Artifact rejection and artifact correction

Blinks and other movements can distort the recorded waveforms. Methods must be in place to reduce or eliminate such artifacts, e.g., by re-recording of segments that are contaminated or software algorithms for artifact correction that can eliminate some of the waveform distortions. It is important to note that the artifact rejection algorithm used can affect the appearance of the resulting mfERG waveform. Consequently, when applying an artifact rejection procedure after the recording, care should be exercised to ensure that clinically important aspects of the waveforms are not being modified. If such procedures are applied, it should be mentioned in the report. The procedures used for processing of the raw signals should be verified, e.g., by reference to the equipment-specific documentation supplied by the manufacturer.

##### Spatial averaging

To reduce noise and to smooth waveforms, analysis software typically allows the averaging of the response from each stimulus element with a percentage of the signal from the neighboring elements. While this spatial averaging can help optimise mfERG signals in noisy records, it is important to minimize or eliminate the causes of noise prior to recording where possible (see “*Artifacts in mfERG recordings”*). Spatial averaging may obscure small, local changes or the borders of regions of dysfunction. Thus, it should be used with care and specified when reporting results. If used, the contribution of the neighbors of any hexagon should in total not exceed the contribution of the local hexagon.

##### Signal extraction/kernels

The standard response is the first-order kernel. Higher-order kernels, particularly the second-order kernel, are reported occasionally and used in special applications. Such responses do not constitute a standard mfERG for routine clinical purposes.

## Clinical protocol

### Patient preparation

#### Pupils

The pupils should be fully dilated, and pupil size noted.

#### Patient positioning

Patients should sit comfortably in front of the screen. A common physiological artifact is from muscle activity and care must be taken to ensure optimal relaxation of facial and neck muscles; a chin and/or headrest may also be helpful. Topical anesthesia may help improve patient comfort and SNR if high noise levels persist. The pupil should be centered in the corneal electrode ring when using contact lens electrodes. The appropriate viewing distance will vary with screen size, in order to control the area (visual angle) of retina being stimulated. If spectacles or trial frames are used, care must be taken to avoid blocking the eccentric stimulus elements.

#### Fixation monitoring

Stable central fixation is essential. Thus, fixation should be monitored, preferably using monitoring instrumentation available on some units, e.g., cameras that allow visualization of the pupil or fundus. When this option is not available, careful direct observation may be employed to assess the stability of fixation. The records and 3D plot of patients suspected of poor fixation due to reduced acuity or poor cooperation should be examined carefully for signs of eccentric fixation. For measures to address eccentric fixation, see “Artifacts in mfERG recordings.”

#### Refraction

Although there is some evidence that mfERGs are unaffected by moderate blurring of the retinal image in healthy individuals, at least when within ± 3 diopters, eyes should be corrected for optimal acuity at the viewing distance taking the patient’s accommodative status into account. Lenses can be placed in a trial frame or holder positioned in front of the eye. On some commercial instruments, a manual adjustment of the viewing optics is possible. It must be avoided that the rim of the lens, trial frame or lens holder blocks the view onto the stimulus and thus creates an apparent scotoma. It should be recognized that refractive correction also affects image size, an effect that becomes significant as the refractive error increases. Consequently, using consistent correction for repeat testing of a patient will optimize serial comparisons of recordings.

#### Monocular versus binocular recording

Time can be saved by recording from both eyes simultaneously, if binocular alignment can be expected. In the case of strabismus, monocular recordings must be performed. Patients with latent strabismus (heterophoria) may fail to align their two eyes onto fixation. In addition, some patients may not sustain adequate convergence onto the stimulus at near, particularly as accommodation is impeded by mydriasis. Traces should be inspected to identify potential artifacts requiring monocular repeat recordings (see “*Artifacts in mfERG recordings”*).

#### Adaptation

##### Pre-exposure to light

The patient should be in ordinary room illumination before testing. Examinations such as indirect ophthalmoscopy and fundus photography should be avoided for at least 15 min prior to mfERG testing. As near as is practical, the pretest light exposure should be the same for all mfERG tests.

##### Room illumination

To avoid peripheral dark adaptation and to maintain a similar level of light adaptation across the retina, moderate or dim room lights should be on and should ideally produce illumination close to that of the stimulus screen. Illumination should be the same for all recordings and the same as for control recordings, and care should be taken to keep any bright light sources and reflections from the lens surface out of the patient’s direct view.

### Duration of recording

A total recording time of at least 4 min for 61 element arrays, or 8 min for 103 element arrays, is recommended, although recording times might be adjusted by experienced laboratories to achieve stable waveforms. The overall recording time may be divided into shorter segments (e.g., 15–30 s) to allow the patient to rest between runs and blink and also to allow for discarding a poor recording segment (from noise, movement, blinking or other artifacts) and repeating them without losing prior data.

## MfERG interpretation and reporting

### MfERG interpretation

The primary clinical application is the detection of changes in mfERG signals to identify damage to discrete retinal regions, in particular, the macula, paramacula or localized eccentric areas of the posterior pole [1]. These clinical evaluations require the examination of the responses to recognize and quantitatively assess where signals are reduced in amplitude or delayed in timing relative to other retinal locations and relative to reference values. For this purpose, the interpretation of mfERGs is performed according to several steps:

1. The mfERG trace arrays of each eye are inspected before processes such as filtering and averaging are preformed to verify the waveforms for technical quality and to check for significant artifacts (see “*Artifacts in mfERG recordings”*).
2. After verification of technical quality, the topography of signals within the trace array is inspected to characterize the location(s), spatial extent and severity of abnormalities.
3. Abnormal signals are quantified in terms of amplitude and peak time measures (see “*mfERG reporting”*). Amplitudes and peak times from groups of responses may be compared to corresponding control values. Grouping of responses may not always be appropriate, e.g., if abnormalities are focal.
4. Additional analysis may involve 3D representations and ring response plots or ring ratios (see “*mfERG reporting”*) to help detect, characterize or monitor dysfunction, although ring averages may not be appropriate if abnormalities lack radial asymmetry.

### MfERG reporting

Standardization of mfERG reporting is critical to the goal of having comparable data worldwide. MfERG reports should follow the details below and should include waveforms, as trace arrays and ring averages if appropriate, and amplitude and peak time measures. Field view can be used for better comparison to visual fields and retinal view for better comparison to fundus images. They can be complemented by other regional averages, 3D-plots and reference ranges. Reports should include other critical information such as the equipment manufacturer, type of recording electrode, length of recording (m-sequence), scaling ratio of stimulus array, use of spatial averaging and artifact correction procedures and comments on any problems during the recording session that might affect reliability and interpretation, such as cooperation, eye movements, head tilt, poor fixation, media opacities, pseudophakia and poor refractive correction.

#### Amplitude measures

The standard measurement for mfERG amplitude and timing is the amplitude measured from the trough of N1 to the peak of P1, and the peak time of P1, respectively (Fig. 2C). In some cases, the amplitude and timing of N1 and the P1:N1 amplitude ratio may be of interest, but these measurements are not part of this standard.

In addition to the standard amplitude and timing measures of the mfERG traces, commercial software typically provides additional measures of the overall amplitude and timing of the mfERG traces, e.g., assessing the overall response waveform by applying response shifting, response stretching, scalar product or root-mean-square functions. These approaches are beyond the scope of this standard.

#### Waveform display and interpretation

**Fig. 3:**
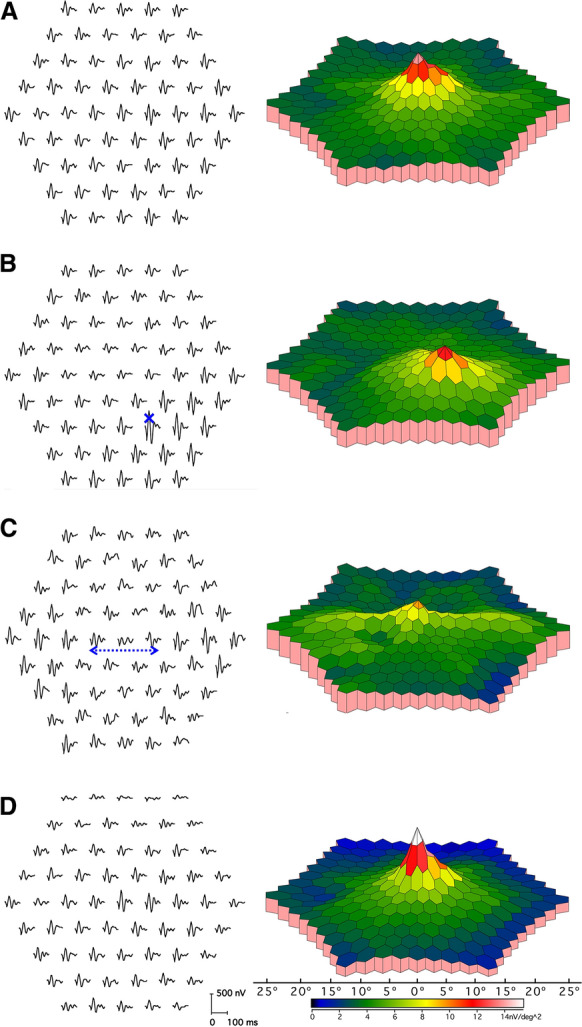
Sample mfERG recording to a 61-element array from a healthy control and illustrations of induced artifacts. Trace arrays (left column) and 3D-response density plots (right column) are depicted in field view (left eye). **A** Control (“normal”) recording for reference. **B** Eccentric fixation. To mimic eccentric fixation, the target was positioned in the lower right visual field (blue cross), where, as a consequence the responses were largest, while they were smallest in the stimulus center. **C** Unstable fixation. To mimic unstable fixation, the participant shifted the gaze between the three central horizontal hexagons (blue dashed line). This created a response reduction in the stimulus center and an enlargement for the lateral hexagons. **D** Positioning error/rim artifact. The view was obscured by the rim of the trial frame, resulting in attenuation of eccentric superior and superior-temporal responses

Trace arrays. The trace array (Figs. 2A and 3, first column) is the basic mfERG display and should always be included in the report of the clinical results. It depicts the original mfERGs projected to their corresponding location in the visual field or retina. This projection can be visualized according to the eccentricity of corresponding hexagonal stimulus elements, or individual responses can be displayed to appear equidistant for presentation purposes. In addition to showing topographic response variations, these arrays allow for an assessment of the quality of the records and the presence of artifacts, which is critical for judging the validity of any suspected deviation from normal. For this purpose, trace lengths of 100 ms or more should be used for these displays to support the detection of interference from line frequency and/or kernel overlap. Critical information such as the mode of display (field view or retinal view) and the dimensions of the stimulus field (degrees) should be provided. Calibration marks must accompany all trace arrays or graphs. This will enable comparisons among patients or within a patient on sequential visits.

Ring and other regional averages*.* Groups of responses from the trace arrays can be averaged for successive rings from center to periphery (ring averages), quadrants, hemiretinal areas, normal and abnormal local areas of interest for comparison between eyes or with reference data. Averaging responses within rings around fixation is particularly useful when studying patients with diseases that produce dysfunction with approximate radial symmetry. In such cases, ring averages should be included in the report of clinical results. To obtain the average response per hexagon, the responses from the hexagons in each ring are summed and subsequently divided by the number of hexagons in the ring (Fig. 2C). For the healthy retina, the ring average will be roughly constant across eccentricities because the stimulus hexagons are scaled to provide comparable response amplitudes. Amplitudes and peak times can be determined from the ring averages for comparison with reference data (see “Reference values (normal ranges)”) if appropriate. The ratios of these ring values to one another can support the detection of abnormalities such as parafoveal loss, because the ratios between rings are relatively stable across the healthy retina. However, clinics will need to establish reference values for the ratios they wish to use. Ring 1 has typically the most variable response among control subjects and, being based on a single response, is not the result of averaging and thus most prone to noise intrusions. Consequently, if ring ratios are used, it is not advised to use ring 1 for the normalization. A caveat is that significant focal changes may be obscured by ring and other averaging, and trace arrays must always be checked for evidence of localized or radially asymmetric abnormalities.

Topographic 3D response density plots. Visualization of a 3D response density plot (Figs. 2B and 3A) can be used to give a topographical overview of the signal strength per unit area of retina (normalization relative to the area of the stimulus patch). Importantly, caution must be exercised when interpreting these plots. First, data about the waveforms are lost. Thus large, but abnormal, or delayed responses can produce normal 3D plots and information relating to specific N1 and P1 components is lost. Second, a central peak in the 3D plot is likely to appear without any physiological retinal signal (see “*Artifacts in mfERG recordings”*). To avoid issues caused by area normalization, as an alternative to the 3D response density plots, 3D plots can be based on non-normalized amplitude measures. Third, the appearance of the 3D plot from a given recording is dependent on how the local amplitude is measured and on data interpolation and filtering. For these reasons, 3D plots should not be used without the simultaneous display of the trace array.

Reference values (“normal” ranges). Reference data from healthy controls should be laboratory-specific and specific for all stimulus and recording conditions, including the type of electrode. Variations in recording equipment and methods make the use of data from other sources inappropriate. Because electrophysiological data are not necessarily described by a normal (Gaussian) distribution, laboratories should report median values rather than means and determine boundaries of normality. The mfERG, like the full-field ERG, may be smaller in amplitude in older individuals and in those with highly myopic eyes so that age and refractive error may be important in the evaluation.

## Artifacts in mfERG recordings

Multifocal ERG recordings can be compromised by artifacts from various sources. Common types of artifacts include line frequency interference, eye movements, eccentric fixation, positioning errors, central peak artifact and waveform distortions from averaging, smoothing, artifact rejections. Careful inspection of the trace arrays is essential to identify such artifacts and to guarantee correct interpretation of the recordings. To support this process, reports should indicate any problems with the recording that might affect reliability and interpretation, such as media opacities, pseudophakia, insufficient refractive correction, blocked view, unstable fixation and high frequency of blinking. Repeat recordings, e.g., with monocular fixation or with adjusted trial frames, may be required to exclude artifactual causes of an abnormal mfERG.

### Line frequency interference

Line (or mains) interference (50 or 60 Hz, depending on country) can be caused by poor electrode contacts that cause unbalanced impedances, or by insufficient grounding and ambient sources of electric noise. Such interference can alter recordings and can usually be identified by inspection of the traces, if they are sufficiently long to comprise several artifact cycles, i.e., at least 100 ms. This interference should not occur under optimal conditions and the presence of strong line interference indicates poor recording conditions, which may affect the validity of the results.

Solution: The line interference should be reduced during the recording by improving electrode contact, grounding, electrical shielding or reducing the area between electrode cables. Removal after the recording using digital filters is possible, but not the preferred solution, since the mfERG has relevant power in that frequency range.

### Eccentric fixation

Eccentric fixation causes systematic alterations of the trace arrays (Fig. 3B). Response maxima are shifted away from the center, such that central responses can appear depressed. The valid results and interpretations cannot be obtained.

Solution: Recordings need to be repeated with correct central fixation. This may be achieved by using fixation targets that are optimized for low vision. Monitoring and encouragement may aid compliance, particularly in children, and steps taken to avoid or minimize patient fatigue. Binocular recordings may assist to identify systematic fixation errors, e.g., caused intentionally, but are only possible in patients without ocular misalignment. The use of unscaled hexagons is not part of the standard mfERG method, but may yield useful information, in conjunction with appropriate reference values, if fixation errors persist.

### Eye movements

Large eye movements and blinks may interfere with the recording and produce drifts and amplifier saturation. Unstable fixation associated with smaller eye-movements reduces localization and can merge responses associated with adjacent hexagons (Fig. 3C). This can reduce the central response and may mask local retinal damage. Focal reduction over the nasal retina may correspond with the optic disk and blind spot, providing an aid to determine fixation quality in some subjects. The absence of attenuation corresponding to the blind spot can be due to poor fixation, but there is inter-subject variability such that the blind spot is not always evident. Given the dimensions of the stimulus array, the blind spot is unlikely to coincide with an entire single hexagon in every subject, especially for low element numbers, and stray light may also be a factor.

Solution: Recordings contaminated with noise and amplifier saturation should be discarded. Re-recording should be accompanied by careful instructions to the patient. Unstable fixation should be noted in the protocol and taken into consideration during the interpretation of the results.

### Positioning errors

Poor positioning or misalignment of the patient, refractive lens frame or recording contact lens with the display may partly block the stimulus and cause mfERG alterations that are not associated with disease (Fig. 3D).

Solution: Recordings need to be repeated with improved positioning of patient and optics. Blocking by the lens frame may be prevented by use of a high-diameter refractive correction lenses placed close to the eye.

### Artifactual central peak

**Fig. 4:**
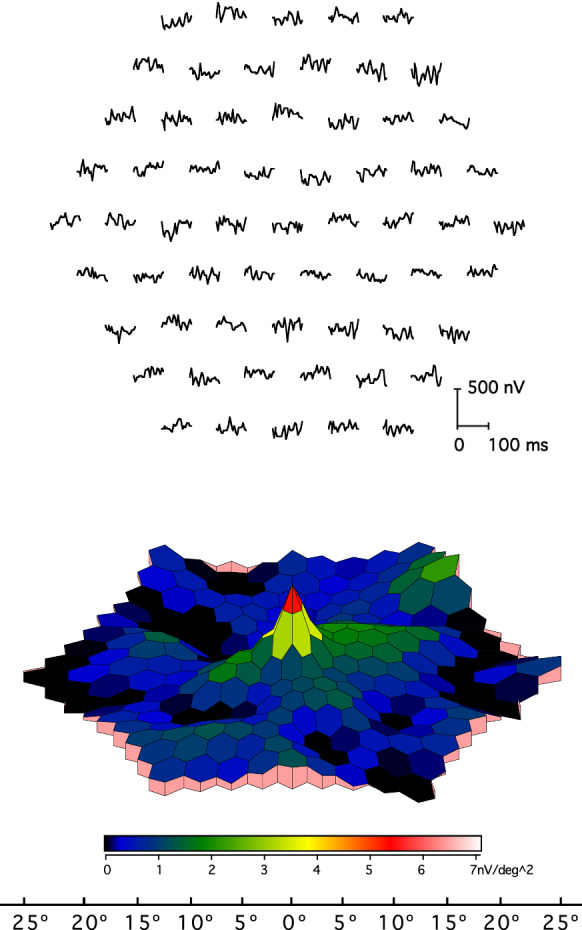
Artifactual central peak. An example of a 61-element “mfERG recording” from a healthy control while the monitor was switched off (trace array and 3D-response density plots). No central response is evident in the trace array, but there is a central peak in the corresponding 3D plot, due to an automated scaling of signals relative to the area of a stimulus element. Noise associated with each hexagon is approximately similar, resulting in increased noise density for smaller hexagons. Note the difference in response density scaling compared with Fig. 3

When scaled arrays are used for stimulation, a central peak may be observed in the 3D response density plots in the absence of physiologically derived mfERGs (Fig. 4). The 3D “response density” plots scale signals (including noise) relative to stimulus area, and this can result in a potentially misleading central peak due to noise.

Solution: Prior to interpretation, the trace arrays must be inspected, to determine the quality and presence of mfERG waveforms. The scaling must be checked.

### Averaging, smoothing, artifact correction

Spatial and temporal averaging, filtering, smoothing and artifact correction can be applied to mfERG traces to reduce noise. These procedures will alter the appearance of the responses.

Solution: Trace arrays with and without such processing must be inspected. If such processing is unavoidable, this should be acknowledged in the report and responses interpreted with appropriate caution.

## Future directions

Future updates of this standard will aim to further unify mfERG recording conditions for better worldwide comparability. This is likely to address sequence length requirements and the advantage of using > 4095 steps, and comparability of the luminance requirements across display types by taking both luminance and flash duration into account.

## Abbreviations

CRT: Cathode ray tube
ERG: Electroretinogram
ISCEV: International Society for Clinical Electrophysiology of Vision
TFT-type LCD: Thin-film-transistor-type liquid crystal display
OLED: Organic light-emitting diode
mfERG: Multifocal electroretinogram
